# eTEP Access Evolving Variants

**DOI:** 10.3389/jaws.2025.15621

**Published:** 2025-10-15

**Authors:** Jorge Daes

**Affiliations:** Minimally Invasive Surgery, Clinica Portoazul, Barranquilla, Colombia

**Keywords:** eTEP technique, eTEP variants, retromuscular repair, preperitoneal repair, PeTEP approach

## Introduction

Originally developed to address the limitations of traditional TEP for inguinal hernias [1], eTEP quickly demonstrated its broader applicability across the abdominal wall. Its versatility lies in its ability to address hernias in any location, its direct retromuscular or preperitoneal access, its flexible port placement, and, when needed, its ability to extend the extraperitoneal space via eTEP-PCS-TAR towards the psoas, cephalad to the central tendon of the diaphragm, or caudally into the inguinal region [2]. Most importantly, eTEP has facilitated the application of modern abdominal wall reconstruction principles, particularly by relocating mesh from an intraperitoneal to an extraperitoneal plane, thereby avoiding the sequelae well documented with the former approach [3].

After its initial use for inguinal, EHS M3, and select Spigelian hernias, two additional eTEP approaches were described:

The eTEP preperitoneal (pretransversalis) approach is accessed directly lateral to the semilunar line and is ideal for EHS L4 hernias [4].
The eTEP Rives-Stoppa approach is a minimally invasive version of the traditional Rives-Stoppa repair and is arguably a form of component separation [5].
Early exploration of the extraperitoneal space should also acknowledge the eMILOS approach—an ingenious hybrid technique that uses the hernia site, usually the umbilicus, to access the space [6].

Since then, multiple refinements have been developed, including:

• eTEP hybrid (various authors)
• eTEP retrorectus “from below” (Mnouskin, Vergara) [7, 8].
• eTEP preperitoneal “from below” (Valenzuela) [9].
• Cranial eTEP preperitoneal (Yu, Deed, García-Ureña, and others) [10].
• Carolina’s eTEP (Sacco, Heniford, and others): one side retromuscular, the other extraperitoneal/preperitoneal [11].
• eTEP precostal approach [12].

These variants do not compete; they complement one another, allowing surgeons to tailor the extraperitoneal approach to each patient. Modern hernia surgeons should understand the indications, advantages, and limitations of each option and acknowledge that extensive data is still needed to confirm their usefulness in daily clinical practice.

## Highlights of Variant Applications

### eTEP Rives–Stoppa

The eTEP Rives–Stoppa approach is distinct in that it provides substantial medialisation. It is essential for achieving closure under physiological tension when a favourable Carbonell rule is met [13], and it is the first step when anterior or posterior component separation is required. Sneider et al. reported that retrorectus dissection alone can contribute up to 41% of the maximum obtainable medialisation in combined component-separation techniques [14]. In this sense, the eTEP Rives–Stoppa approach offers an advantage over the other eTEP accesses and remains the best-studied variant.

### eTEP Access Lateral to the Semilunar Lines

This is ideal for lumbar (EHS-L4) hernias and is also used in the triple neurectomy approach to create space. It accesses the pretransversalis or preperitoneal layer by going under the lateral muscles at the linea semilunaris. This can be achieved through blunt dissection, using an Optiview trocar, or under direct vision via an intraperitoneal 5 mm port. The space itself can be developed either bluntly or using a balloon dissector [4].

### eTEP Hybrid

This is useful in cases involving skin dystrophy, mesh densely adhering to the bowel, an incarcerated or irreducible bowel, lateral complex hernias, or the challenges faced by surgeons with limited experience of minimally invasive anterior fascial closure. It combines the safety of addressing these issues with the advantages of the MIS approach, as was evident during the early development of MIS colon surgery.

### eTEP Performed Directly “From Below”

Mnouskin described a direct “from below” entry that eliminates the need for an additional superior port to create space. Although the entry is preperitoneal, dissection proceeds retromuscularly. This limits dissection to small midline ventral hernias (umbilical/supraumbilical), with or without diastasis, and addresses the frequent criticism that traditional eTEP is “too much” for minor defects.

### eTEP Preperitoneal “From Below” (PeTEP)

This approach is particularly beneficial for small to medium-sized midline defects, with or without diastasis. It appeals to surgeons who emphasise preserving the integrity of the posterior rectus sheath and neurovascular bundles, thereby theoretically preventing bulging [9]. However, it has limitations: it can be harder to replicate due to peritoneal tears, it does not provide medialisation, and it relies on a thin peritoneal layer for adhesion prevention, which remains a concern.

### Cranial eTEP Preperitoneal Approach

This is preferable for small central defects to avoid extensive dissection—even in combination with inguinal hernias — but standard port placement precludes diastasis repair.

### Carolina’s eTEP

This is defined as an eTEP access with one side retrorectus and the other preperitoneal [11]. It may be helpful for:

• Moderate midline hernias with or without moderate diastasis when the retrorectus space is insufficient for ideal mesh placement; by avoiding PCSTAR solely for mesh extension, it reduces complexity.
• Unilateral parastomal hernias, where spanning the defect is more straightforward.
• Patients at risk of cosmetic bulging after Rives–Stoppa, such as young, thin, and muscular patients and athletes, who may experience muscle atrophy from neurovascular bundle trauma, as demonstrated recently [15].

### eTEP Precostal Approach

The precostal approach [12] has become a reliable access for the majority of ventral repairs—particularly laparoscopic ones—by (i) precisely demarcating the semilunar lines for optimal lateral port placement, (ii) facilitating a high crossover, (iii) providing a vantage point that often renders a traditional fourth port unnecessary, and (iv) enabling a suturing vector that is not constrained to “distal-to-camera.”

## Adjunct Strategies

### Robotic-Assisted eTEP

Robotics has eased the transfer of skills to complex cases. Three-dimensional vision, wristed instruments in confined spaces, and easy suturing—especially along the “ceiling”—suit this approach well [16].

### Enhanced Imaging

Enhance imaging has helped to clarify the anatomy relevant to safely extending the extraperitoneal space, especially cranially. One example is understanding how the transversus abdominis transitions into diaphragmatic fibres, which are separated by a fatty rim at the costal margin—this is knowledge that makes cephalad extension towards the central tendon safer [17]. Another example is that, when extending the dissection cranially, it should start 6 cm below the xiphoid process in the preperitoneal space and continue beneath the retrosternal fat pad to avoid damaging neural and diaphragmatic fibres and causing excessive bleeding.

### Other Selected Strategies

Selected strategies can be combined with eTEP for specific indications. Botulinum toxin, fascial-tension devices, and the Inan inverting suture [18] can be used for anterior fascial closure (this is effective in preventing the unsightly upper-midline ridge).

### Afterword

By recognising the contributions of the many surgeons who have refined eTEP access, we now have a versatile toolkit that can be matched to the needs of each patient. Each variant has a role, and knowing the nuances helps to optimise outcomes in abdominal wall reconstruction. Above all, every repair should be planned as if it were the patient’s last repair. While the instinct to “save planes for the future” may be prudent, it should be applied cautiously—never at the expense of achieving the best long-term result possible today.
